# Supplementary material for: AI prediction of cardiovascular events using opportunistic epicardial adipose tissue assessments from CT calcium score
Source: ArXiv. 2024 Jan 29:arXiv:2401.16190v1. Preprint. [Version 1] (PMC10862931)
Supplement: Supplement 1 [file NIHPP2401.16190v1-supplement-1.pdf]

Supplemental Materials:
S.1 Detailed feature engineering
Fat-omics involves precise measurements of heart structure and epicardial adipose tissue (EAT). The heart is equally divided into four axial slabs (Positional Quartiles (PQ)) from the top (PQ1) to the bottom (PQ4). EAT Hounsfield unit quartiles (HQ), ranging from 1 to 4, categorize HU values into bins: HQ1 includes values from -190 to -150, HQ2 from -150 to -110, HQ3 from -110 to -70, and HQ4 from -70 to -30. Spherical regions (SR) consisted of equidistant radial shells from the outside (SR1) to the inside (SR4) of the heart. We divided the thickness measurements into four fixed histogram bins (each 8mm wide).

1. ***Structural features***
    - **Total_SAC_Volume_Cm3** (total pericardial sac volume in cm^3)
    - **WHV** (whole heart volume: pericardial sac volume – EAT volume)
    - **PrincipalAxisLength_max** (major principal axis length of the pericardial sac)
    - **PrincipalAxisLength_min** (minor principal axis length of the pericardial sac)
    - **PrincipalAxisLength_med** (intermediate principal axis length of the pericardial sac)
2. ***EAT features***
- **Total_Volume_Cm3** (EAT volume in cm^3)
- **Total_Normalized_EAT** (EAT volume/pericardial sac volume)
- **Total_HU<<stats>>** (<<mean, median, max, min, kurtosis, skewness>> of the HU of EAT)
- **Thickness_<<stats>>** (<<mean, median, max, min, std>> of the EAT thickness)
- **NormThickness_<<stats>>** (<<mean, median, max, min, std>> of the EAT thickness divided by the corresponding radius in the same direction)
- **Thickness_bin<<number>>_Pro (**probability of fixed thickness bins**)**
- **<<stats>>HU_PQ<<number>>** (<<mean, median, max, min, std, kurtosis, skewness>> of the HU in each positional quartile <<number>> [1-4])
- **Vol_PQ<<number>>** (volume of EAT in cm^3 in each position quartile <<number>> [1-4])
- **<<stats>>HU_HQ<<number>>** (<<mean, median, max, min, std, kurtosis, skewness>> of EAT HU in each HU bin HQ <<number>> [1-4])
- **PixelCount_HQ<<number>>** (number of voxels of EAT in HQ <<number>> [1-4])
- **Probability_HQ<<number>>** (probability of EAT Voxels with HU in HQ <<number>> [1-4])
- **Vol_HQ<<number>>** (EAT volume in each HQ <<number>> [1-4])
- **Pro_HQ<<HU range>>** (probability of EAT voxels in each HQ <<number>> [1-4])
- **SR<<number>>_Pro_<<HU range>>** (probability of EAT voxels in HU ranges <<HU range>> ([-190, -170], [-170, -150], [-150, -130], [-130, -110], [-110, -90], [-90, -70], [-70, -50], [-50, -30]) in each spherical region <<number>>[1-4])

S.2 Radiomics feature analyses

We provided additional detailed analyses into fat-omics features that correlate with the risk of major adverse cardiovascular events (MACE). These analyses included a range of EAT features, stratified by spherical regions (SR), Hounsfield Unit (HU) bins, and fixed thickness bins, to uncover the most significant predictors of MACE.

Figure S1 expanded on the predictive power of EAT volume from different spherical regions (SR), with special emphasis on the outermost layer (SR1), which demonstrated the highest significance in MACE prediction. This aligned with the anatomical fact that SR1 are proximal to the coronary arteries, also subjecting to pericoronary adipose analysis. Table S2 delved into the EAT HU normalized distributions, examining the probability of voxel volumes within specific HU ranges. The highest bin (Pro_50_30) still showed the highest significance among all bins. Table S3 presented analysis of EAT fixed thickness bins, offering insights into the distribution of thickness across predefined bins and their relationship to MACE, thus revealing the importance of EAT thickness heterogeneity in cardiovascular risk assessment. Table S4 consisted of all selected EAT features by cox elastic net, underscoring the intricate relationship between EAT characteristics and cardiovascular health. All high-risk features mentioned in the main text were included in final fat-omics model.

Variables	c-index	AIC	HR[95%CI]	p_value
EAT_vol	0.53	2345.81	1.2 (1.06:1.36)	0.0046**
**Vol_SR1**	**0.56**	**2341.32**	**1.26 (1.11:1.43)**	**<0.001*** **
Vol_SR2	0.54	2345.04	1.21 (1.07:1.37)	0.0029**
Vol_SR3	0.53	2347.57	1.17 (1.03:1.32)	0.013*
Vol_SR4	0.53	2351.07	1.1 (0.975:1.24)	0.12

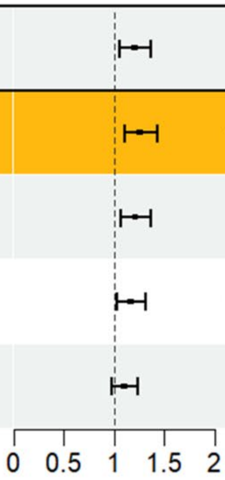

Table S1: MACE prediction from EAT volume, including the contributions from different shells sub-regions. Each row presents a univariate Cox proportional hazards model for the respective feature, including the hazard ratio, AIC, and corresponding p-value. The total EAT volume was divided into four quartiles shells from outer to inner (SR1-SR4), where the Vol_SR1 represents to EAT volume at the most outer shell. Similar as slabs sub-regions analyses in Table 3, EAT in the most outer shell of the heart is the most significant with a C-index of 0.56. This aligns with the anatomical fact that SR1 are proximal to the coronary arteries, also subjecting to pericoronary adipose analysis.

Variables	c-index	AIC	HR[95%CI]	p_value
Pro_190_170	0.53	2351.99	1.07 (0.955:1.2)	0.24
Pro_170_150	0.52	2351.27	1.09 (0.968:1.24)	0.15
Pro_150_130	0.5	2352.30	1.07 (0.939:1.21)	0.32
Pro_130_110	0.48	2353.25	1.01 (0.885:1.15)	0.9
Pro_110_90	0.54	2350.23	0.886 (0.772:1.02)	0.083
Pro_90_70	0.54	2344.82	0.822 (0.719:0.938)	0.0037**
Pro_70_50	0.48	2353.26	0.995 (0.872:1.13)	0.94
Pro_50_30	0.55	2350.26	1.13(1.001:1.29)	0.05*

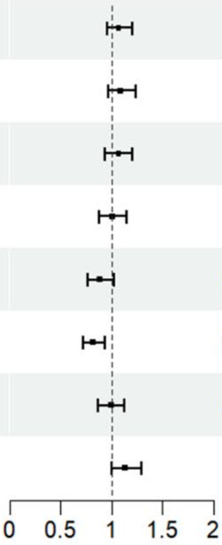

Table S2: Analysis of EAT HU normalized distribution. In additional to just using the volumes of tissue within each range, we normalized histograms and obtained a probability of being within a range of HU values. Probability of EAT corresponding to HU ranges are analyzed where Pro_190_170 corresponds to the probability of EAT having HU values within -190 to -170 HU. Results were only a little poorer than obtained in Table 4, while the highest bin still showed significance.

Variables	c-index	AIC	HR[95%CI]	p_value
thickness_bin41_Pro	0.49	2353.03	0.968 (0.851:1.1)	0.63
thickness_bin42_Pro	0.48	2353.18	1.02 (0.895:1.16)	0.77
thickness_bin43_Pro	0.52	2350.73	1.1 (0.988:1.22)	0.083
**thickness_bin44_Pro**	**0.53**	**2347.79**	**1.19 (1.05:1.35)**	**0.0059****

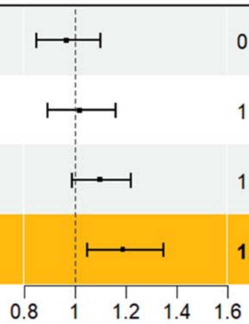

Table S3: Fixed histogram bin analysis of EAT thickness. Similar as HU, we delved deeper by dividing the thickness measurements into four fixed histogram bins (each 8mm wide), determined by the spread of thickness observed across all patients. Probability of thickness are analyzed where thickness_bin44_Pro indicating the probability of thickness between 24 to 32mm. As expected, the largest thickness bin is the most significant feature, consistent with our findings in Fig. 4 (long tail thickness outlier is more significant).

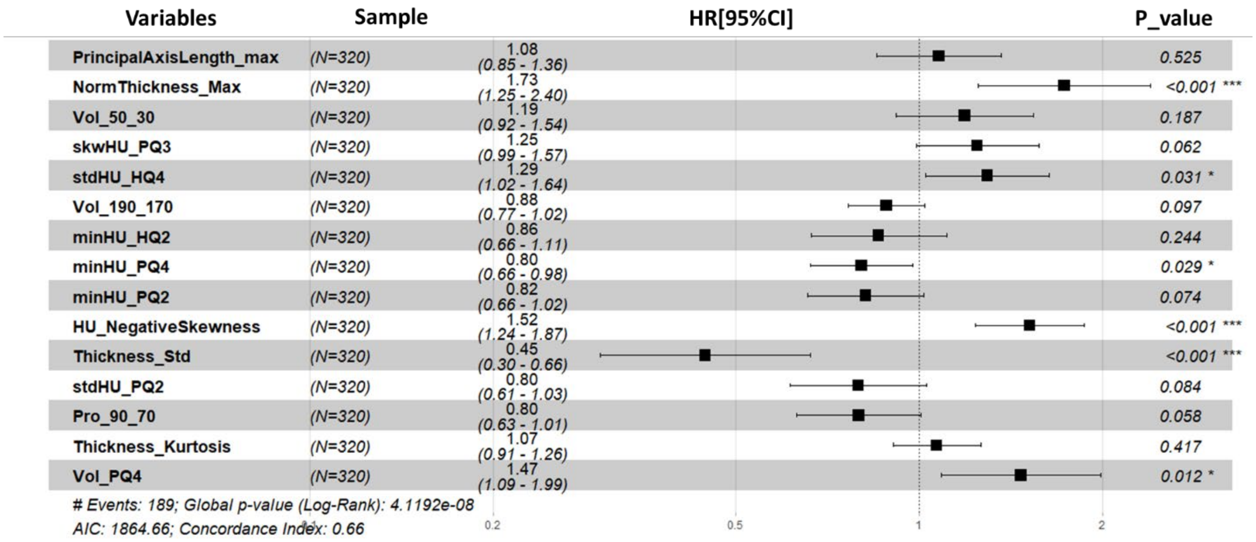

Variables	Sample	HR[95%CI]	P_value
PrincipalAxisLength_max	(N=320)	1.08 (0.85 - 1.36)	0.525
NormThickness_Max	(N=320)	1.73 (1.25 - 2.40)	<0.001 ***
Vol_50_30	(N=320)	1.19 (0.92 - 1.54)	0.187
skwHU_PQ3	(N=320)	1.25 (0.99 - 1.57)	0.062
stdHU_HQ4	(N=320)	1.29 (1.02 - 1.64)	0.031 *
Vol_190_170	(N=320)	0.88 (0.77 - 1.02)	0.097
minHU_HQ2	(N=320)	0.86 (0.66 - 1.11)	0.244
minHU_PQ4	(N=320)	0.80 (0.66 - 0.98)	0.029 *
minHU_PQ2	(N=320)	0.82 (0.66 - 1.02)	0.074
HU_NegativeSkewness	(N=320)	1.52 (1.24 - 1.87)	<0.001 ***
Thickness_Std	(N=320)	0.45 (0.30 - 0.66)	<0.001 ***
stdHU_PQ2	(N=320)	0.80 (0.61 - 1.03)	0.084
Pro_90_70	(N=320)	0.80 (0.63 - 1.01)	0.058
Thickness_Kurtosis	(N=320)	1.07 (0.91 - 1.26)	0.417
Vol_PQ4	(N=320)	1.47 (1.09 - 1.99)	0.012 *

Events: 189; Global p-value (Log-Rank): 4.1192e-08
AIC: 1864.66; Concordance Index: 0.66

Table S4: List of all selected EAT features in EAT-Omics.

We started with 148 EAT features and performed maximum relevance minimum redundancy (MRMR) to exclude highly correlated features, resulting 50 uncorrelated EAT features. Among all preselected features, 15 dominant features were selected by cox elastic net. We performed cox proportional hazard analysis on those 15 selected features. Corresponding hazard ratio and p-value were listed in the table. All significant features introduced previously were included in this table.
